# Demand for family planning satisfied with modern methods among sexually active women in low- and middle-income countries: who is lagging behind?

**DOI:** 10.1186/s12978-018-0483-x

**Published:** 2018-03-06

**Authors:** Fernanda Ewerling, Cesar G. Victora, Anita Raj, Carolina V. N. Coll, Franciele Hellwig, Aluisio J. D. Barros

**Affiliations:** 1 International Center for Equity in Health, Federal University of Pelotas, Pelotas, Brazil; 2 Postgraduate Program in Epidemiology, Federal University of Pelotas, Pelotas, Brazil; 3 Center on Gender Equity and Health, University of California San Diego, San Diego, USA

**Keywords:** Family planning, Contraception, Socioeconomic factors, Health equity

## Abstract

**Background:**

Family planning is key for reducing unintended pregnancies and their health consequences and is also associated with improvements in economic outcomes. Our objective was to identify groups of sexually active women with extremely low demand for family planning satisfied with modern methods (mDFPS) in low- and middle-income countries, at national and subnational levels to inform the improvement and expansion of programmatic efforts to narrow the gaps in mDFPS coverage.

**Methods:**

Analyses were based on Demographic and Health Survey and Multiple Indicator Cluster Survey data. The most recent surveys carried out since 2000 in 77 countries were included in the analysis. We estimated mDFPS among women aged 15–49 years. Subgroups with low coverage (mDFPS below 20%) were identified according to marital status, wealth, age, education, literacy, area of residence (urban or rural), geographic region and religion.

**Results:**

Overall, only 52.9% of the women with a demand for family planning were using a modern contraceptive method, but coverage varied greatly. West & Central Africa showed the lowest coverage (32.9% mean mDFPS), whereas South Asia and Latin America & the Caribbean had the highest coverage (approximately 70% mean mDFPS). Some countries showed high reliance on traditional contraceptive methods, markedly those from Central and Eastern Europe, and the Commonwealth of Independent States (CEE & CIS). Albania, Azerbaijan, Benin, Chad and Congo Democratic Republic presented low mDFPS coverage (< 20%). The other countries had mDFPS above 20% at country-level, yet in many of these countries mDFPS coverage was low among women in the poorest wealth quintiles, in the youngest age groups, with little education and living in rural areas. Coverage according to marital status varied greatly: in Asia & Pacific and Latin America & the Caribbean mDFPS was higher among married women; the opposite was found in West & Central Africa and CEE & CIS countries.

**Conclusions:**

Almost half of the women in need were not using an effective family planning method. Subgroups requiring special attention include women who are poor, uneducated/illiterate, young, and living in rural areas. Efforts to increase mDFPS must address not only the supply side but also tackle the need to change social norms that might inhibit uptake of contraception.

**Electronic supplementary material:**

The online version of this article (10.1186/s12978-018-0483-x) contains supplementary material, which is available to authorized users.

## Plain English summary

Universal access to sexual and reproductive health is key for sustainable development. However, the family planning literature is mainly restricted to women that are married or in a union, so that non-partnered women (especially adolescents) remain overlooked. We provide further and timely insight on the status of global demand for family planning satisfied with modern methods (mDFPS) among all sexually active women. Using data from 77 low- and middle-income countries, we showed that almost half of the women in need of family planning were not using a modern contraceptive method. According to marital status, mDFPS varied greatly: in Asia & Pacific and Latin America & the Caribbean mDFPS was higher among married women; the opposite was found in West & Central Africa and CEE & CIS countries. We also mapped the groups of women who are lagging in terms of mDFPS and in need of special attention in terms of programs and policies. This group includes five countries where overall mDFPS was below 20% (Albania, Azerbaijan, Benin, Chad and Congo Democratic Republic) and, in many other countries, women in the poorest wealth quintiles, in the youngest age groups, with little education and living in rural areas. Efforts to increase mDFPS must be directed not only to increase the availability of contraception, but also towards the empowerment of women and changes in social norms that might inhibit uptake of modern contraception.

## Background

Universal access to sexual and reproductive health, inclusive of family planning, is a key objective for sustainable development, and “leaving no one behind” is one of the main features of the Sustainable Development Goals (SDGs) launched in 2015 by the United Nations. Reproductive health is explicitly mentioned in goal 3 on good health and wellbeing, but may also be considered as part of goal 5, which aims at gender equality and women’s empowerment [1, 2]. Provision of safe, effective and affordable modern contraceptive methods is central to achieve high levels of demand for family planning satisfied (DFPS) and address women’s sexual and reproductive health needs [3, 4]. The optimal use of modern contraceptive methods can help prevent unintended pregnancies and induced abortions in low- and middle-income countries (LMICs) [5], and will directly contribute to improved maternal and child health outcomes [6–10]. Family planning also has potential to reduce poverty worldwide by improving educational and economic outcomes for women [3, 11].

Although DFPS is increasing worldwide [8, 12, 13], coverage remains unacceptably low in many LMICs, with 20–58% of women at reproductive age not using contraception despite their desire to delay or limit pregnancy [14]. Large gaps in demand for family planning satisfied with modern methods (mDFPS) between countries still remain. Furthermore, within-country socioeconomic disparities in access to modern contraception persist [15, 16]. Lower levels of DFPS are reported for women who are younger, poorer and living in rural areas [14]. Lack of access to information or services, as well as the stigma attached to contraceptive use due to social norms and expectations surrounding early marriage and motherhood, lead women to not use contraception even when they desire to avoid pregnancy [14, 17, 18]. Qualitative evidence also indicates that female disapproval of modern family planning methods is influenced by their limited understanding of potential side effects [14]. Acceptance of modern contraceptive methods tend to be more common among women with a higher educational level [14].

Efforts to achieve universal mDFPS require the assessment of within-country inequalities and identification of low-coverage population subgroups [16]. In this context, disaggregated analyses according to key stratifiers are essential [1]. An important limitation is that most of the family planning literature is based on married women; expanding this focus to all sexually active women may provide further insights on who is being left behind [10, 19–22]. Our objective was to identify which groups of sexually active women are not being reached by family planning programs, both at national and subnational levels, to inform the improvement and expansion of programmatic efforts to narrow the gaps in mDFPS.

## Methods

We used data from the most recent (post-2000) Demographic and Health Surveys (DHS) or Multiple Indicator Cluster Surveys (MICS) from each country. These are publicly available, nationally representative, cross-sectional surveys conducted in LMICs. DHS and MICS samples include around 15,000 and 10,000 households respectively, drawn with multistage cluster sampling (usually two-stage). Besides other information on maternal and child health outcomes and interventions, these surveys regularly gather information on reproductive health and family planning. Both DHS and MICS present similar questionnaires, methodology and sampling strategy, which ensures the comparability of results. We identified 95 surveys, from which we excluded 18 (see Additional file 1: Table S1) that only contained information on reproductive health for women who were married or in a union. Thus, 77 surveys remained in our analyses.

Our main indicator was the proportion of women using contraception among those in need of contraception. This indicator is also referred to in the literature as family planning coverage [23], or other similar denominations. Differently from other indicators commonly used in the field, such as unmet need for contraception for which the denominator includes all women, this is a coverage indicator as the denominator is restricted to the sexually active women in need of contraception. We estimated demand for family planning satisfied with any contraceptive (DFPS) as well as with modern methods only (mDFPS) among sexually active women aged 15–49 years. Women were considered sexually active either if they were in a union or reported a sexual intercourse in the 4 weeks before the survey. MDFPS was calculated according to the 2012 update of the indicator definition [24]. According to this definition, women in need of contraception are defined as those who are fecund and do not want to become pregnant within the next 2 years, or who are unsure about whether or when they want to become pregnant. Women are considered infecund if they (1) are married for five or more years, never used contraception and had no children in the past 5 years; (2) said that they cannot get pregnant; (3) are menopausal, had a hysterectomy or never menstruated; or (4) had last period more than 6 months ago and are not postpartum amenorrheic. Pregnant women with a mistimed or unwanted pregnancy are also considered in need of contraception. Modern contraceptive methods were defined as technological products or medical procedures that affect natural reproduction [25]. According to this definition, modern methods include contraceptive pills, condoms (male and female), intrauterine device (IUD), sterilization (male and female), injectables, hormone implants, patches, diaphragms, spermicidal agents (foam/jelly), and emergency contraception.

We also present mDFPS coverage according to type of contraception, coded as: (1) modern, short acting methods including contraceptive pills, injectables, condoms, diaphragms, patch, spermicidal agents and emergency contraception; (2) modern, long acting methods, including IUD and hormone implants; and (3) modern, permanent methods, that comprise male and female sterilizations. Reliance on modern methods (% modern) was calculated as the ratio between the mDFPS and the DFPS. Traditional methods include lactational amenorrhea, abstinence, rhythm or calendar methods, and withdrawal.

Several analyses are presented by world region, according to the UNICEF classification: West & Central Africa, Eastern & Southern Africa, Central and Eastern Europe & the Commonwealth of Independent States (CEE & CIS), South Asia, East Asia & Pacific and Latin America & the Caribbean. Mean mDFPS was estimated for each world region according to marital status (women in a union vs not in a union). Aggregation of mDFPS by world region was done by averaging the mDFPS of all countries with available information in each world region. These are unweighted means, so each country has the same weight, regardless of the countries’ population size.

To identify countries, areas and subgroups of women who are being left behind, we arbitrarily defined low coverage as being below 20%. Analyses were stratified by woman’s age (15–17 years; 18–19 years; 20–49 years old), education (none; primary; secondary or higher), literacy (yes; no); union, defined as being currently married or in a union (yes; no), wealth quintiles, based on the asset index included in the survey datasets (Q1 being the poorest and Q5 the richest quintile), area (urban; rural) and region of residence in the country (classified according to the surveys), and religion defined in DHS as the woman’s religion, and in MICS as the religion of the household head. The logic of the age grouping was not risk, but social vulnerability. Our approach aimed at highlighting how the adolescents fare in terms of family planning compared to adult women.

The surveys are based on complex samples and all estimates considered the sample design, including clusters, strata and sample weights. All analyses were conducted using Stata (StataCorp. 2013. Stata Statistical Software: Release 13. College Station, TX: StataCorp LP).

## Results

Overall, mDFPS ranged from 13.5% (in Albania) to 89.5% (in Cuba), with a global unweighted mean coverage of 52.9% (see Table 1). South Asia and Latin America & the Caribbean presented the highest average mDFPS, around 70%, followed by East Asia & Pacific, Eastern & Southern Africa, CEE & CIS, and West & Central Africa. The mean mDFPS in West & Central Africa was 32.9%, with countries ranging from 17.6% to 51.5%. Eleven surveys were available for the Middle East & North Africa in the study period, but none had information for sexually active women who were not in union. Table 1 also presents the mDFPS subdivided by type of contraceptive used and its last column indicates the proportion of the DFPS (with any contraceptive method) that was due to modern contraceptive methods. Diverse countries from CEE & CIS have low reliance on modern contraception (< 50% modern). Most woman in need of contraception residing in countries such as Albania, Armenia, Azerbaijan, Bosnia and Herzegovina and Kosovo are still relying on traditional contraception. Congo and Congo Democratic Republic also showed lower shares of DFPS with modern methods. Zimbabwe, Swaziland, Uzbekistan, Lesotho, Namibia, Indonesia, Barbados, Saint Lucia, Lao People’s Democratic Republic and Bhutan are the top ten countries for the use of modern, short term contraception. In Kazakhstan, Kyrgyzstan, Vietnam, Tajikistan and Mongolia more than a third of the women relied on long-acting contraception. Permanent contraception was common among women in need of contraception in eleven countries, including three from South Asia (India, Nepal and Bhutan) and eight from Latin American & Caribbean (Dominican Republic, Colombia, Costa Rica, Nicaragua, Panama, Belize, Honduras, and Cuba). In these countries, more than one quarter of the couples included a partner who had been sterilized.

**Table 1:** List of surveys with the Demand for family planning satisfied (DFPS) and DFPS with modern methods (mDFPS) estimates by type of contraceptive method used

					Modern method			
Region	Country	Year	Total DFPS	Modern DFPS	Short acting^a^	Long acting^b^	Permanent^c^	% modern^d^
Central and Eastern Europe and the Commonwealth of Independent States (CEE & CIS)	Albania	2008	84.2	13.5	8.8	1.1	3.6	16.1
	Armenia	2010	80.2	39.4	25.0	14.0	0.4	49.1
	Azerbaijan	2006	69.3	19.3	6.4	12.4	0.6	27.9
	Belarus	2012	90.2	77.0	52.3	20.3	4.3	85.4
	Bosnia and Herzegovina	2011	81.2	29.0	22.7	6.1	0.3	35.8
	Kazakhstan	2010	83.4	81.4	26.3	53.1	1.9	97.5
	Kosovo	2013	90.1	22.3	14.4	6.8	1.0	24.7
	Kyrgyzstan	2012	66.3	61.5	18.9	39.7	2.9	92.7
	Macedonia	2005	28.7	21.7	15.3	4.9	1.5	75.7
	Moldova	2012	87.5	66.3	30.9	29.0	6.4	75.8
	Montenegro	2013	60.9	47.6	37.3	10.2	0.1	78.2
	Serbia	2010	91.5	48.8	44.8	3.8	0.2	53.3
	Tajikistan	2012	55.0	50.9	13.2	36.6	1.2	92.6
	Ukraine	2012	92.2	70.6	52.0	17.4	1.2	76.5
	Uzbekistan	2006	89.3	83.0	73.0	6.8	3.2	93.0
East Asia & Pacific	Cambodia	2014	81.8	56.1	42.0	9.5	4.5	68.9
	Indonesia	2012	84.4	78.9	64.5	9.9	4.6	93.5
	Mongolia	2013	81.4	73.0	32.8	35.5	4.8	89.7
	Philippines	2013	75.7	51.2	34.7	4.8	11.6	67.6
	Timor-Leste	2009	42.0	39.7	34.1	4.1	1.5	94.5
	Vietnam	2010	95.3	75.0	31.0	38.9	5.0	78.7
	Lao People’s Democratic Republic	2011	78.0	68.0	58.2	2.6	7.3	87.2
Eastern & Southern Africa	Burundi	2010	40.3	32.8	25.6	6.1	1.1	81.4
	Comoros	2012	38.8	27.8	23.2	3.0	1.5	71.6
	Ethiopia	2011	52.6	50.2	42.6	6.8	0.9	95.4
	Kenya	2014	76.4	70.4	49.1	17.1	4.1	92.1
	Lesotho	2014	77.0	76.4	70.9	3.5	2.0	99.3
	Madagascar	2008	67.0	48.7	43.7	3.1	1.9	72.6
	Malawi	2013	75.3	74.0	47.8	13.3	12.9	98.2
	Mozambique	2011	34.7	33.9	32.9	0.5	0.5	97.9
	Namibia	2013	79.3	77.3	69.6	1.4	6.3	97.5
	Rwanda	2014	72.3	63.5	49.5	12.1	1.9	87.9
	Swaziland	2010	84.6	83.0	74.4	3.0	5.7	98.1
	Tanzania, United Republic of	2010	62.6	50.7	39.8	5.0	5.9	80.9
	Uganda	2011	48.0	41.5	32.3	4.9	4.4	86.6
	Zambia	2013	67.9	62.0	50.4	9.1	2.5	91.4
	Zimbabwe	2014	88.0	87.5	75.0	11.4	1.1	99.5
Latin America & Caribbean	Barbados	2012	75.1	70.9	60.2	4.6	6.1	94.5
	Belize	2011	75.4	71.4	42.9	2.0	26.5	94.8
	Bolivia	2008	75.5	43.4	25.4	10.3	7.7	57.4
	Colombia	2010	91.5	83.2	33.0	12.0	38.1	90.9
	Costa Rica	2011	88.1	86.8	51.1	2.8	33.0	98.4
	Cuba	2014	90.3	89.5	37.4	27.2	24.9	99.0
	Dominican Republic	2014	84.2	82.9	33.2	4.4	45.3	98.5
	Guyana	2014	53.5	51.6	35.9	10.5	5.2	96.4
	Haiti	2012	48.5	44.1	39.5	2.5	2.1	90.9
	Honduras	2011	87.2	75.9	42.1	7.9	25.9	87.0
	Nicaragua	2001	82.4	79.2	41.2	7.6	30.4	96.1
	Panama	2013	77.3	74.5	41.9	3.0	29.5	96.4
	Peru	2012	90.6	62.1	49.8	3.1	9.2	68.6
	Saint Lucia	2012	76.0	72.3	59.0	4.5	8.8	95.1
	Suriname	2010	69.7	69.2	52.7	2.5	13.9	99.3
	Trinidad and Tobago	2006	61.0	56.3	26.4	18.7	11.2	92.3
South Asia	Bhutan	2010	85.8	85.6	55.0	4.9	25.6	99.7
	India	2005	81.9	69.9	12.2	2.5	55.2	85.4
	Nepal	2011	64.3	55.9	22.9	3.2	29.7	86.8
West & Central Africa	Benin	2011	30.4	18.9	15.6	3.1	0.3	62.3
	Burkina Faso	2010	41.1	38.4	29.2	8.8	0.4	93.2
	Cameroon	2011	53.6	35.5	32.8	1.8	1.0	66.2
	Central African Republic	2010	36.3	24.6	23.7	0.4	0.4	67.6
	Chad	2014	20.3	18.1	13.7	3.6	0.8	89.4
	Congo	2011	73.8	36.4	36.0	0.2	0.2	49.3
	Côte d’Ivoire	2011	41.9	29.8	29.1	0.6	0.1	71.0
	Gabon	2012	58.5	40.3	39.3	0.2	0.8	68.9
	Gambia	2013	27.1	24.5	20.1	2.7	1.7	90.4
	Ghana	2014	48.0	38.9	26.1	9.8	2.9	81.0
	Guinea	2012	24.3	20.3	19.2	0.8	0.2	83.4
	Guinea-Bissau	2006	38.4	29.5	23.6	5.0	0.9	76.8
	Liberia	2013	41.0	38.6	34.0	4.3	0.4	94.1
	Mali	2012	29.0	27.8	19.5	7.9	0.4	95.8
	Niger	2012	46.5	40.8	39.1	1.2	0.5	87.9
	Nigeria	2013	52.3	35.1	29.9	4.2	1.0	67.2
	Sao Tome and Principe	2014	55.0	51.5	45.1	5.6	0.8	93.6
	Senegal	2015	48.3	43.7	30.0	12.9	0.8	90.4
	Sierra Leone	2013	47.9	45.3	35.8	8.6	0.9	94.4
	Togo	2013	39.2	34.1	24.5	9.2	0.4	87.1
	Congo Democratic Republic	2013	43.6	17.0	13.9	1.6	1.5	39.0

^a^Modern, short acting contraceptive methods: pill, injectable, condom, diaphragm, patch, spermicide (foam/jelly), and emergency contraception

^b^Modern, long acting contraceptive methods: intrauterine device (IUD) and implants

^c^Modern, permanent contraception: male and female sterilization

^d^The share of DFPS with modern contraceptive methods (% modern) was calculated as the ratio between the mDFPS and the DFPS

**Fig. 1:**
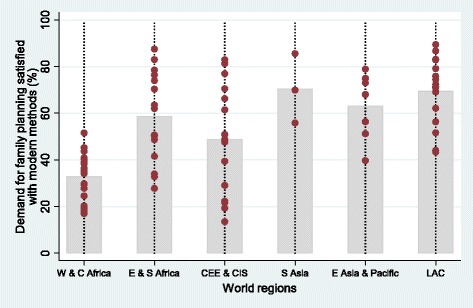
Demand for family planning satisfied with modern contraceptive methods by country and world region. Notes: The bars represent the average mDFPS by world region and the dots represent the coverage in each country. World regions are West & Central Africa; Eastern & Southern Africa; Central, Eastern Europe and the Commonwealth of Independent States; South Asia; East Asia & Pacific; and Latin America & the Caribbean. The estimates consider all sexually active women

Between-country inequalities in mDFPS are large in all regions, as shown in Fig. 1, where each dot represents one country. CEE & CIS and Eastern & Southern Africa were the regions with the largest spreads across the countries, presenting gaps of 69.5% and 59.7%, respectively, comparing the countries with highest and lowest coverage in each region. West & Central Africa had the largest number of countries with data, the lowest mean, and was one of the most homogeneous, with only one country with coverage above 50%.

Women in need of contraception that were not in a union were much more likely to use modern contraception in West and Central Africa and CEE & CIS. However, the opposite trend was observed in Latin America & Caribbean, South Asia and East Asia & Pacific (Table 2).

**Table 2:** Mean demand for family planning satisfied with modern methods (mDFPS), % modern^a^ and mean mDFPS according to marital status by region of the world

Region	Number of countries	Mean mDFPS	% modern^a^	Mean mDFPS	
				Not in union	In a union
West & Central Africa	21	32.9	78.6	46.6	30.2
Eastern & Southern Africa	15	58.8	90.2	58.0	58.5
CEE & CIS	15	48.9	65.0	57.9	46.8
South Asia	3	70.5	90.6	58.8	70.5
East Asia & Pacific	7	63.2	82.9	35.3	63.7
Latin America & Caribbean	16	69.6	91.0	65.2	70.4
Total	77	52.9	81.7	54.3	51.9

^a^The share of DFPS with modern contraceptive methods (% modern) was calculated as the ratio between the mDFPS and the demand for family planning satisfied with any contraceptive method

**Fig. 2:**
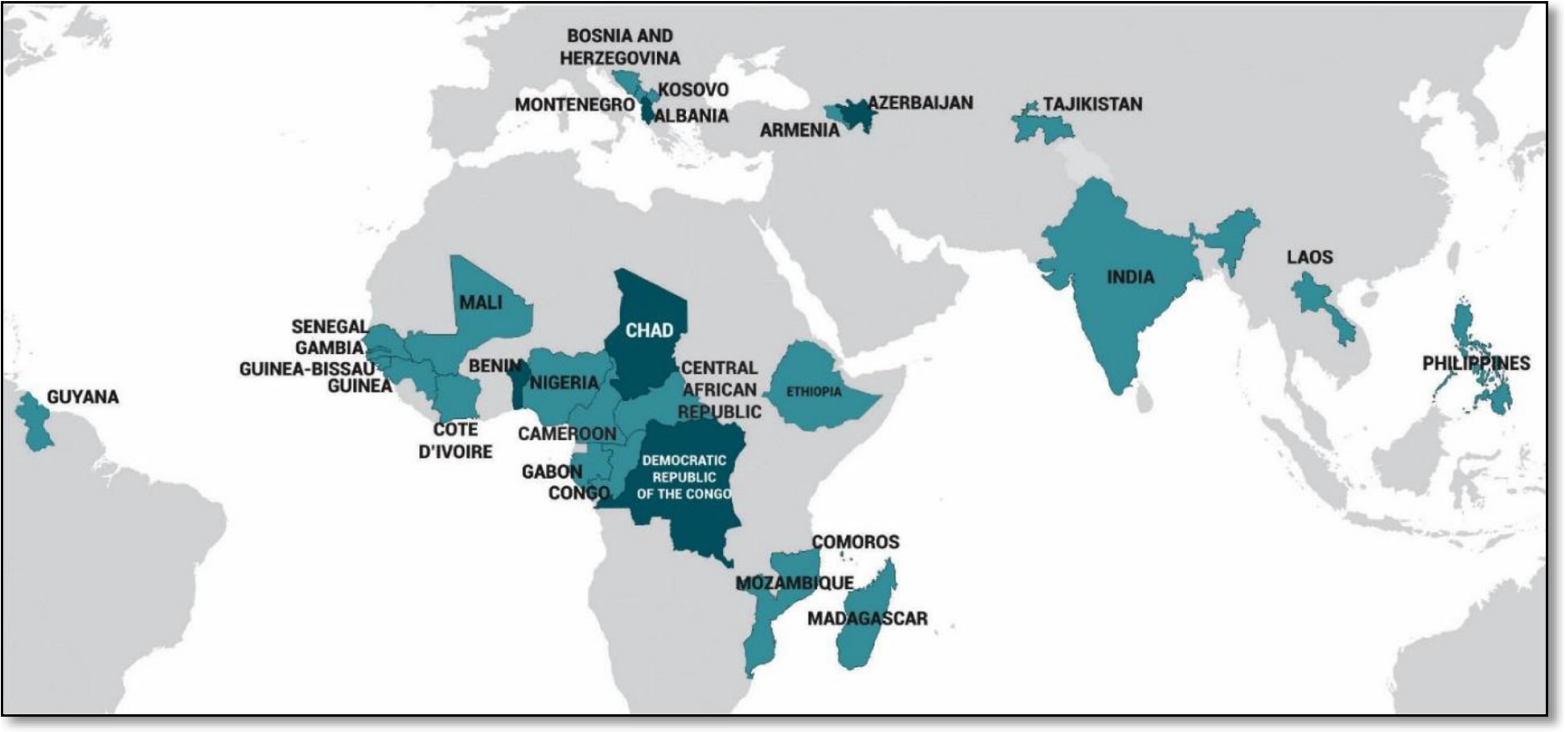
Map of countries and subgroups with demand for family planning satisfied with modern contraceptive methods (mDFPS) below 20%. Note: The darker colored countries have an overall mDFPS below 20%. The lighter colored countries have an overall mDFPS above 20%, yet these countries have at least one subgroup with mDFPS below 20%

Figure 2 presents a map of all countries and subgroups with mDFPS below 20%, taken as an indication of low coverage. Five countries were found to have low mDFPS: Albania, Azerbaijan, Benin, Chad and Congo Democratic Republic. Another eleven countries presented at least one subgroup with mDFPS below 20%. As shown in Table 3, these subgroups tended to include woman who are poor, uneducated/illiterate, young and living in the rural areas. We also identified specific religious groups: Islam in Central African Republic and Guinea; Animism in Cameroon, Congo and Mali; and other religions (which comprises different small religious groups in each country) in Cote d’Ivoire, Ethiopia, Guinea-Bissau and Mali. In Guinea and Guinea Bissau, married women had low coverage, whereas in Lao People’s Democratic Republic, this was the case for unmarried women. Coverage levels in each country and subgroup with low mDFPS are presented in Additional file 1: Table S2.

**Table 3:** Demand for family planning satisfied with modern contraceptive methods (mDFPS) below 20% by country and subgroups

Country (Survey year)	Younger	Rural area	Less educated	Illiterate	Religion	Poorer	Union
Armenia (2010)	●						
Bosnia and Herzegovina (2011)			●	●		●	
Central African Republic (2010)	●	●	●	●	ISLAM	●	
Cameroon (2011)			●	●	ANIMISM	●	
Comoros (2012)			●			●	
Congo (2011)			●		ANIMISM	●	
Côte d’Ivoire (2011)					OTHER	●	
Ethiopia (2011)					OTHER		
Gabon (2012)			●				
Gambia (2013)	●	●	●	●		●	
Guinea (2012)	●	●	●	●	ISLAM	●	YES
Guinea-Bissau (2006)		●	●	●	OTHER	●	YES
Guyana (2014)	●						
India (2005)	●	●					
Kosovo (2013)		●	●	●		●	
Lao People’s Democratic Republic (2011)							NO
Madagascar (2008)	●						
Mali (2012)	●				ANIMISM OTHER	●	
Montenegro (2013)			●				
Mozambique			●			●	
Nigeria (2013)			●			●	
Philippines (2013)	●						
Senegal (2015)	●						
Tajikistan (2012)	●						

## Discussion

This is a comprehensive overview of demand for family planning satisfied in low and middle-income countries, using recent survey data. Family planning, despite all technological advances and the variety of methods available, remains a controversial issue [14]. On average, only 52.9% of women who need contraception are using a modern method. This means that almost half of the women who are fertile but not willing to get pregnant are failing to be reached by effective family planning strategies. Great differences in mDFPS were found between and within countries. Low mDFPS (below 20%) was found in Albania, Azerbaijan, Benin, Chad and Congo Democratic Republic and, in many other countries, among women in the poorest wealth quintiles, in the youngest age groups, with little education and living in rural areas.

The lowest average mDFPS coverage was found in West & Central Africa. A substantial difference in mDFPS was found between sexually active women in a union and those who are not in a union, with much higher coverage in the latter group. The region reports high rates of child marriage [26, 27] in addition to many of its countries having the lowest women’s empowerment levels in Africa [28]. This suggests that efforts must be directed not only at the supply side – including provision of contraceptives through appropriate delivery channels - but also at reducing child marriage and at increasing woman’s empowerment through changes in social norms that might inhibit uptake of contraception (including among married woman) [29–32]. West and Central African countries have also been identified in a previous publication by Choi and colleagues as those with the largest gap between projected and required changes to achieve the SGD family planning benchmark by 2030 [33].

Low mDFPS coverage is found among countries where overall contraceptive use is low, or where women mainly rely on traditional methods [9, 34]. A substantial proportion of women report relying on withdrawal and periodic abstinence in some countries from CEE & CIS. Individual effectiveness of the traditional contraceptive methods is generally lower than the efficacy of the modern methods [35]. Thus, the low fertility rates found in countries with high levels of contraceptive use, but a large reliance on traditional methods, can be a result of traditional contraceptive being effective at the population level. In some countries, especially those in the CEE & CIS region, induced abortion can play a role as well [36]. Thus, the challenge of offering effective family planning options to a population is not only about making modern contraceptives easily available, but also making them acceptable, and in many situations, transitioning the use of traditional to modern methods.

The countries mapped in Fig. 2, all of which have low mDFPS, deserve special attention from global level stakeholders, as well as from national health managers and policy makers. With such low coverage at national level, these countries need urgent and comprehensive initiatives to increase family planning uptake. The subgroups highlighted in Table 3 are not surprising as they tend to include women who are poor, uneducated/illiterate, young, and living in the rural areas. The reasons behind low coverage vary across countries and regions. The literature indicates that the most widespread reasons are related to opposition from partners or others, concerns about side effects and low perceived risk of getting pregnant due to infrequent sexual activity [37, 38]. Lack of access to contraceptives is not one of the most common barriers for use [37, 39]. In some social contexts there is intense pressure for a pregnancy shortly after marriage and a large number of children may also be perceived as a welcome help in rural settings [17, 38]. Even though some religions condemn the use of contraceptives, it did not come up in our analysis as an important determinant of mDFPS (Table 3). In some countries, Islam, Animism and other religions appear among the low coverage subgroups, but wealth, education and age are much more consistent markers.

Another interesting determinant was whether or not the woman was in a union. In some regions, sexually active women outside a union were much more likely to use a modern method, given that most are not willing to get pregnant. In other regions, however, women in union presented a much higher mDFPS. These paradoxical findings suggest that the availability of contraceptives may not be the primary barrier, as access may be affected by social norms and barriers [40]. In some places, married women are expected to have children and therefore not to use contraception; on the other hand, unmarried women may have restricted access to contraception due to taboos against sex outside marriage [22, 41]. Unmarried women are, on average, younger than those in a union, which may also constitute a barrier.

There is an intense debate about the classification of the contraceptive methods as modern or traditional [25]. The World Health Organization classifies some fertility awareness methods, such as the Standard Days, TwoDay method, sympto-thermal and basal body temperature as modern contraceptives [42]. However, many surveys do not get into enough detail to differentiate rhythm from these modern forms of fertility awareness methods. Also, in LMICs settings, with large proportions of women with very low educational and empowerment levels, it seems unlikely that these methods will have high effectiveness.

Previous studies on family planning coverage in LMICs are mostly limited to women who are married or in union, therefore leaving aside a substantial number of women (unmarried and sexually active) who need family planning [33, 43]. As some countries do not collect data on reproductive health for unmarried women, some regions may be underrepresented in our study, especially the Middle East and North Africa where no country had available data. Also, many unmarried women may not feel comfortable to report having already had sexual activity, which could potentially bias the estimates. Another limitation is the subjectivity involved in the women’s perception of wanting the current or a next pregnancy, as this response may change in the course of the pregnancy or depending on circumstances in life.

One of the main strengths of the study is our comparable estimates for mDFPS for all sexually active women regardless their marital status at a global scale, using data from both DHS and MICS surveys. This is a significant contribution to the current understanding in the field. We additionally used eight sociodemographic stratifiers, which allowed us to identify subgroups that are lagging in terms of demand for family planning satisfied, addressing within-countries inequalities. This is critical to track progress towards achieving the target of universal access to sexual and reproductive health care services of the post-2015 SDGs and reach the benchmark of demand satisfied with modern methods by 2030 [19, 33]. The increased availability and frequency of national demographic and health surveys, coupled with growing emphasis on disaggregated statistics during the SDG era will allow analyses such as ours to be repeated periodically. This will allow the description of time trends at national and subnational level, allowing the monitoring of equity gaps, the identification of success stories as well as of persistent challenges.

## Conclusion

We showed that in LMICs almost half of the women in need of family planning were not using a modern contraceptive method. Demand for family planning satisfied with modern methods is highly variable globally, within regions and within countries. Albania, Azerbaijan, Benin, Chad and Congo Democratic Republic presented mDFPS below 20%. Frequently, women in the poorest wealth quintiles, in the youngest age groups, with little education and living in rural areas also presented low mDFPS. In Asia & Pacific and Latin America & the Caribbean mDFPS was higher among married women compared to the unmarried ones whereas the opposite was found in West & Central Africa and CEE & CIS countries. Efforts to increase mDFPS must be directed not only to increased availability through appropriate delivery channels, but also towards empowerment of women and changes in social norms that might inhibit uptake of contraception.

## Additional file

Additional file 1: **Table S1.** Countries without information on reproductive health among unmarried, sexually active women, excluded from the analyses. **Table S2.** Demand for family planning satisfied with modern contraceptive methods (mDFPS) below 20% by country and subgroups. (DOCX 24 kb)

## References

[1] Raj A, McDougal L (2017). Leaving no one behind: can the family planning estimation tool help?. Lancet Glob Health.

[2] Watkins K (2014). Leaving no one behind: an agenda for equity. Lancet.

[3] Prata N, Fraser A, Huchko MJ, Gipson JD, Withers M, Lewis S, Ciaraldi EJ, Upadhyay UD. Womens empowerment and family planninig: a review of the literature. J Biosoc Sci. 2017;49(6):713–43. 10.1017/S0021932016000663 PMC5503800 28069078

[4] Machiyama K, Casterline JB, Mumah JN, Huda FA, Obare F, Odwe G, Kabiru CW, Yeasmin S, Cleland J (2017). Reasons for unmet need for family planning, with attention to the measurement of fertility preferences: protocol for a multi-site cohort study. Reprod Health.

[5] Bellizzi S, Sobel HL, Obara H, Temmerman M (2015). Underuse of modern methods of contraception: underlying causes and consequent undesired pregnancies in 35 low- and middle-income countries. Hum Reprod.

[6] Ahmed S, Li Q, Liu L, Tsui AO (2012). Maternal deaths averted by contraceptive use: an analysis of 172 countries. Lancet.

[7] Social franchising: a blockbuster to address unmet need for family planning and to advance toward the FP2020 goal. Glob Health Sci Pract. 2015;3:147–8. 10.9745/GHSP-D-15-00155 PMC4476853 26085012

[8] Schivone GB, Blumenthal PD (2016). Contraception in the developing world: special considerations. Semin Reprod Med.

[9] United Nations (2015). Trends in contraceptive use worldwide.

[10] Cleland J, Machiyama K (2015). Unmet need for family planning: past achievements and remaining challenges. Semin Reprod Med.

[11] Canning D, Schultz TP (2012). The economic consequences of reproductive health and family planning. Lancet.

[12] Alkema L, Kantorova V, Menozzi C, Biddlecom A (2013). National, regional, and global rates and trends in contraceptive prevalence and unmet need for family planning between 1990 and 2015: a systematic and comprehensive analysis. Lancet.

[13] New JR, Cahill N, Stover J, Gupta YP, Alkema L (2017). Levels and trends in contraceptive prevalence, unmet need, and demand for family planning for 29 states and union territories in India: a modelling study using the family planning estimation tool. Lancet Glob Health.

[14] Wulifan JK, Brenner S, Jahn A, De Allegri M (2016). A scoping review on determinants of unmet need for family planning among women of reproductive age in low and middle income countries. BMC Womens Health.

[15] Aslam SK, Zaheer S, Qureshi MS, Aslam SN, Shafique K (2016). Socio-economic disparities in use of family planning methods among Pakistani women: findings from Pakistan demographic and health surveys. PLoS One.

[16] Ross J (2015). Improved reproductive health equity between the poor and the rich: an analysis of trends in 46 low- and middle-income countries. Glob Health Sci Pract.

[17] UNFPA. Motherhood in childhood: facing the challenge of adolescent pregnancy: UNFPA; 2013. p. 132.

[18] Parsons J, Edmeades J, Kes A, Petroni S, Sexton M, Wodon Q (2015). Economic impacts of child marriage: a review of the literature. The Review of Faith & International Affairs.

[19] Fabic MS, Choi Y, Bongaarts J, Darroch JE, Ross JA, Stover J, Tsui AO, Upadhyay J, Starbird E (2015). Meeting demand for family planning within a generation: the post-2015 agenda.

[20] Wagstaff A, Bredenkamp C, Buisman LR (2014). Progress on global health goals: are the poor being left behind?. The World Bank Research Observer.

[21] Lowe SM, Moore S (2014). Social networks and female reproductive choices in the developing world: a systematized review. Reprod Health.

[22] Darroch JE, Singh S (2013). Trends in contraceptive need and use in developing countries in 2003, 2008, and 2012: an analysis of national surveys. Lancet.

[23] Barros AJ, Boerma T, Hosseinpoor AR, Restrepo-Mendez MC, Wong KL, Victora CG (2015). Estimating family planning coverage from contraceptive prevalence using national household surveys. Glob Health Action.

[24] Bradley SEK, Croft TN, Fishel JD, Westoff CF (2012). Revising unmet need for family planning: DHS analytical studies no. 25.

[25] Hubacher D, Trussell J (2015). A definition of modern contraceptive methods. Contraception.

[26] Raj A (2010). When the mother is a child: the impact of child marriage on the health and human rights of girls. Arch Dis Child.

[27] UNICEF (2014). Ending child marriage: progress and prospects.

[28] Ewerling F, Lynch JW, Victora CG, van Eerdewijk A, Tyszler M, Barros AJD. The SWPER index for women’s empowerment in Africa: development and validation of an index based on survey data. Lancet Glob Health. 2017;5:e916–e923. 10.1016/S2214-109X(17)30292-9 PMC5554795 28755895

[29] Upadhyay UD, Gipson JD, Withers M, Lewis S, Ciaraldi EJ, Fraser A, Huchko MJ, Prata N (2014). Women's empowerment and fertility: a review of the literature. Soc Sci Med.

[30] OlaOlorun FM, Hindin MJ (2014). Having a say matters: influence of decision-making power on contraceptive use among Nigerian women ages 35-49 years. PLoS One.

[31] Mboane R, Bhatta MP (2015). Influence of a husband's healthcare decision making role on a woman's intention to use contraceptives among Mozambican women. Reprod Health.

[32] Blackstone SR. Women's empowerment, household status and contraception use in Ghana. J Biosoc Sci. 2016:1–12. 10.1017/S0021932016000377 27510983

[33] Choi Y, Fabic MS, Hounton S, Koroma D (2015). Meeting demand for family planning within a generation: prospects and implications at country level. Glob Health Action.

[34] Williamson LM, Parkes A, Wight D, Petticrew M, Hart GJ (2009). Limits to modern contraceptive use among young women in developing countries: a systematic review of qualitative research. Reprod Health.

[35] Polis CB, Bradley SE, Bankole A, Onda T, Croft T, Singh S (2016). Typical-use contraceptive failure rates in 43 countries with demographic and health survey data: summary of a detailed report. Contraception.

[36] Sedgh G, Bearak J, Singh S, Bankole A, Popinchalk A, Ganatra B, Rossier C, Gerdts C, Tunçalp Ö, Johnson BR (2016). Abortion incidence between 1990 and 2014: global, regional, and subregional levels and trends. Lancet.

[37] Barot S (2015). Sexual and reproductive health and rights are key to global development: the case for ramping up investment.

[38] Pinter B, Hakim M, Seidman DS, Kubba A, Kishen M, Di Carlo C (2016). Religion and family planning. Eur J Contracept Reprod Health Care.

[39] Wulifan JK, Brenner S, Jahn A, De Allegri M. A scoping review on determinants of unmet need for family planning among women of reproductive age in low and middle income countries. BMC Womens Health 2015;16:2. 10.1186/s12905-015-0281-3 PMC4714507 26772591

[40] Sedgh G, Hussain R (2014). Reasons for contraceptive nonuse among women having unmet need for contraception in developing countries. Stud Fam Plan.

[41] Kragelund Nielsen K, Nielsen SM, Butler R, Lazarus JV (2012). Key barriers to the use of modern contraceptives among women in Albania: a qualitative study. Reprod Health Matters.

[42] World Health Organization: http://www.who.int/mediacentre/factsheets/fs351/en/. Acessed 3 Jan 2018.

[43] Alkenbrack S, Chaitkin M, Zeng W, Couture T, Sharma S (2015). Did equity of reproductive and maternal health service coverage increase during the MDG era? An analysis of trends and determinants across 74 low- and middle-income countries. PLoS One.

