# Supplementary material for: Demand for family planning satisfied with modern methods among sexually active women in low- and middle-income countries: who is lagging behind?
Source: Reprod Health. 2018 Mar 6;15:42. doi: 10.1186/s12978-018-0483-x (PMC5840731; doi:10.1186/s12978-018-0483-x)
Supplement: Supplementary file 1 — Table S1. Countries without information on reproductive health among unmarried, sexually active women, excluded from the analyses. Table S2. Demand for family planning satisfied with modern contraceptive methods (mDFPS) below 20% by country and subgroups. (DOCX 24 kb) [file 12978_2018_483_MOESM1_ESM.docx]

Table S1. Countries without information on reproductive health among unmarried, sexually active women, excluded from the analyses.

| Region | Country | ISO code | Survey Year | Source^1^ |
| --- | --- | --- | --- | --- |
| CEE & CIS^2^ | Turkmenistan | TKM | 2006 | MICS |
| East Asia & Pacific | Thailand | THA | 2012 | MICS |
| Middle East & North Africa | Algeria | DZA | 2012 | MICS |
| Middle East & North Africa | Djibouti | DJI | 2006 | MICS |
| Middle East & North Africa | Egypt | EGY | 2014 | DHS |
| Middle East & North Africa | Iraq | IRQ | 2011 | MICS |
| Middle East & North Africa | Jordan | JOR | 2012 | DHS |
| Middle East & North Africa | State of Palestine | PSE | 2014 | MICS |
| Middle East & North Africa | Sudan | SDN | 2014 | MICS |
| Middle East & North Africa | Syrian Arab Republic | SYR | 2006 | MICS |
| Middle East & North Africa | Tunisia | TUN | 2011 | MICS |
| Middle East & North Africa | Yemen | YEM | 2013 | DHS |
| South Asia | Bangladesh | BGD | 2014 | DHS |
| South Asia | Maldives | MDV | 2009 | DHS |
| South Asia | Pakistan | PAK | 2012 | DHS |
| West & Central Africa | Mauritania | MRT | 2011 | MICS |

Notes: ^1^ Multiple Indicator Cluster Survey (MICS) and Demographic and Health Survey (DHS)

^2^ Central and Eastern Europe and the Commonwealth of Independent States

Table S2. Demand for family planning satisfied with modern contraceptive methods (mDFPS) below 20% by country and subgroups.

| **Country** | **Survey Year** | **Source^1^** | **Subgroup** | **mDFPS** | **N (unweighted)** |
| --- | --- | --- | --- | --- | --- |
| **Countries with mDFPS below 20% at national level** | | | |  |  |
| Albania | 2008 | DHS | National | 13.5% | 4269 |
| Azerbaijan | 2006 | DHS | National | 19.3% | 3849 |
| Benin | 2011 | DHS | National | 18.9% | 5864 |
| Chad | 2014 | DHS | National | 18.1% | 3734 |
| Congo, Democratic Republic | 2013 | DHS | National | 17.6% | 6529 |
| **Subgroups with mDFPS below 20%** | | |  |  |  |
| Armenia | 2010 | DHS | Age: 18-19 years | 11.0% | 29 |
| Bosnia and Herzegovina | 2011 | MICS | Wealth: 2nd quintile | 18.8% | 435 |
| Bosnia and Herzegovina | 2011 | MICS | Literate: no | 2.9% | 44 |
| Bosnia and Herzegovina | 2011 | MICS | Education: primary | 12.7% | 567 |
| Central African Republic | 2010 | MICS | Wealth: 2nd quintile | 5.9% | 755 |
| Central African Republic | 2010 | MICS | Age: 15-17 years | 17.4% | 220 |
| Central African Republic | 2010 | MICS | Area: rural | 7.9% | 2080 |
| Central African Republic | 2010 | MICS | Education: none | 7.4% | 1267 |
| Central African Republic | 2010 | MICS | Literate: no | 14.4% | 2732 |
| Central African Republic | 2010 | MICS | Wealth: 1st quintile | 5.8% | 601 |
| Central African Republic | 2010 | MICS | Religion: Islam | 15.2% | 405 |
| Central African Republic | 2010 | MICS | Wealth: 3rd quintile | 11.9% | 860 |
| Cameroon | 2011 | DHS | Literate: no | 19.3% | 1910 |
| Cameroon | 2011 | DHS | Wealth: 1st quintile | 10.2% | 603 |
| Cameroon | 2011 | DHS | Education: none | 11.4% | 701 |
| Cameroon | 2011 | DHS | Religion: Animism | 17.9% | 80 |
| Comoros | 2012 | DHS | Wealth: 1st quintile | 19.8% | 340 |
| Congo | 2011 | DHS | Religion: Animism | 15.7% | 33 |
| Congo | 2011 | DHS | Education: none | 19.9% | 411 |
| Congo | 2011 | DHS | Wealth: 1st quintile | 17.5% | 1906 |
| Côte d'Ivoire | 2011 | DHS | Religion: Other | 13.8% | 53 |
| Côte d'Ivoire | 2011 | DHS | Wealth: 1st quintile | 18.4% | 641 |
| Ethiopia | 2011 | DHS | Religion: Other | 18.8% | 34 |
| Gabon | 2012 | DHS | Education: none | 19.6% | 156 |
| Gambia | 2013 | DHS | Wealth: 1st quintile | 15.2% | 481 |
| Gambia | 2013 | DHS | Education: primary | 18.3% | 351 |
| Gambia | 2013 | DHS | Wealth: 2nd quintile | 14.9% | 556 |
| Gambia | 2013 | DHS | Literate: no | 18.0% | 1705 |
| Gambia | 2013 | DHS | Area: rural | 14.8% | 1356 |
| Gambia | 2013 | DHS | Education: none | 18.1% | 1374 |
| Gambia | 2013 | DHS | Age: 15-17 years | 11.0% | 48 |
| Gambia | 2013 | DHS | Age: 18-19 years | 11.4% | 92 |
| Gambia | 2013 | DHS | Wealth: 3rd quintile | 19.3% | 464 |

| **Country** | **Survey Year** | **Source^1^** | **Subgroup** | **mDFPS** | **N (unweighted)** |
| --- | --- | --- | --- | --- | --- |
| Guinea | 2012 | DHS | Age: 18-19 years | 17.2% | 177 |
| Guinea | 2012 | DHS | Wealth: 3rd quintile | 16.0% | 413 |
| Guinea | 2012 | DHS | Wealth: 4th quintile | 19.0% | 596 |
| Guinea | 2012 | DHS | Wealth: 1st quintile | 10.8% | 403 |
| Guinea | 2012 | DHS | Age: 15-17 years | 14.0% | 121 |
| Guinea | 2012 | DHS | Area: rural | 14.1% | 1365 |
| Guinea | 2012 | DHS | Religion: Islam | 18.2% | 2037 |
| Guinea | 2012 | DHS | Education: none | 15.6% | 1556 |
| Guinea | 2012 | DHS | Union: yes | 15.8% | 1977 |
| Guinea | 2012 | DHS | Literate: no | 16.1% | 1859 |
| Guinea | 2012 | DHS | Education: primary | 19.8% | 321 |
| Guinea | 2012 | DHS | Wealth: 2nd quintile | 15.3% | 376 |
| Guinea-Bissau | 2006 | MICS | Wealth: 1st quintile | 5.2% | 338 |
| Guinea-Bissau | 2006 | MICS | Union: yes | 18.6% | 1731 |
| Guinea-Bissau | 2006 | MICS | Area: rural | 10.1% | 1132 |
| Guinea-Bissau | 2006 | MICS | Religion: Other | 19.6% | 30 |
| Guinea-Bissau | 2006 | MICS | Wealth: 2nd quintile | 9.5% | 360 |
| Guinea-Bissau | 2006 | MICS | Literate: no | 18.1% | 1711 |
| Guinea-Bissau | 2006 | MICS | Education: none | 9.8% | 1182 |
| Guinea-Bissau | 2006 | MICS | Wealth: 3rd quintile | 13.5% | 456 |
| Guyana | 2014 | MICS | Age: 15-17 years | 11.8% | 89 |
| India | 2005 | DHS | Age: 15-17 years | 11.6% | 597 |
| Kosovo | 2013 | MICS | Literate: no | 18.4% | 125 |
| Kosovo | 2013 | MICS | Education: none | 18.2% | 57 |
| Kosovo | 2013 | MICS | Education: primary | 17.5% | 115 |
| Kosovo | 2013 | MICS | Wealth: 2nd quintile | 19.2% | 398 |
| Kosovo | 2013 | MICS | Wealth: 1st quintile | 18.9% | 427 |
| Kosovo | 2013 | MICS | Area: rural | 19.9% | 1315 |
| Lao People's Democratic Republic | 2011 | MICS | Union: no | 5.4% | 95 |
| Madagascar | 2008 | DHS | Age: 15-17 years | 19.4% | 401 |
| Mali | 2012 | DHS | Age: 15-17 years | 15.7% | 123 |
| Mali | 2012 | DHS | Religion: Animism | 6.7% | 34 |
| Mali | 2012 | DHS | Religion: Other | 19.7% | 58 |
| Mali | 2012 | DHS | Wealth: 2nd quintile | 16.7% | 536 |
| Mali | 2012 | DHS | Wealth: 1st quintile | 11.4% | 495 |
| Mali | 2012 | DHS | Wealth: 3rd quintile | 17.3% | 573 |
| Montenegro | 2013 | MICS | Education: primary | 16.1% | 132 |
| Mozambique | 2011 | DHS | Wealth: 2nd quintile | 17.6% | 499 |
| Mozambique | 2011 | DHS | Education: none | 18.5% | 921 |
| Mozambique | 2011 | DHS | Wealth: 1st quintile | 11.2% | 389 |
| Nigeria | 2013 | DHS | Education: none | 10.1% | 2338 |
| Nigeria | 2013 | DHS | Wealth: 1st quintile | 6.0% | 1010 |
| Nigeria | 2013 | DHS | Wealth: 2nd quintile | 19.7% | 1455 |
| **Country** | **Survey Year** | **Source^1^** | **Subgroup** | **mDFPS** | **N (unweighted)** |
| Philippines | 2013 | DHS | Age: 15-17 years | 18.1% | 65 |
| Senegal | 2015 | DHS | Age: 15-17 years | 14.5% | 69 |
| Tajikistan | 2012 | DHS | Age: 18-19 years | 13.4% | 40 |

Notes: ^1^ Multiple Indicator Cluster Survey (MICS) and Demographic and Health Survey (DHS)
